# Correction: Facile preparation of a 3D rGO/g-C_3_N_4_ nanocomposite loaded with Ag NPs for photocatalytic degradation

**DOI:** 10.1039/d5ra90078f

**Published:** 2025-06-13

**Authors:** Kesheng Cao, Xueyu Ge, Shuang Li, Zhengshan Tian, Suya Cui, Guijin Guo, Liuqing Yang, Xingwu Li, Yabo Wang, Suzhen Bai, Qian Wei, Wei Liu

**Affiliations:** a School of Chemistry and Environmental Engineering, Henan Province Engineering Technology Research Center of Green Hydrogen & Electrochemical Energy Storage, Pingdingshan University Pingdingshan 467000 China tianzhengshan@163.com; b College of Optical, Mechanical and Electrical Engineering, Zhejiang A&F University Hangzhou 311300 China qweiyk@126.com liuwei@zafu.edu.cn

## Abstract

Correction for ‘Facile preparation of a 3D rGO/g-C_3_N_4_ nanocomposite loaded with Ag NPs for photocatalytic degradation’ by Kesheng Cao *et al.*, *RSC Adv.*, 2025, **15**, 17089–17101, https://doi.org/10.1039/D5RA02399H.

The authors regret that the name of one of the authors (Wei Liu) was shown incorrectly in the original article. The corrected author list is as shown above.

The Royal Society of Chemistry apologises for these errors and any consequent inconvenience to authors and readers.

